# Posterior indirect reduction and pedicle screw fixation without laminectomy for Denis type B thoracolumbar burst fractures with incomplete neurologic deficit

**DOI:** 10.1186/s13018-015-0227-3

**Published:** 2015-05-29

**Authors:** Zhigang Zhang, Guangdong Chen, Jiajia Sun, Genlin Wang, Huilin Yang, Zongping Luo, Jun Zou

**Affiliations:** Department of Orthopaedic Surgery, The First Affiliated Hospital of Soochow University, Suzhou, Jiangsu 215006 China

**Keywords:** Thoracolumbar burst fracture, Posterior approach, Decompression, Neurologic recovery

## Abstract

**Purpose:**

The aim of this study is to evaluate the efficacy of posterior indirect reduction and pedicle screw fixation without laminectomy for the treatment of Denis type B thoracolumbar burst fractures with incomplete neurologic deficit.

**Methods:**

From March 2008 to May 2012, 36 consecutive patients of Denis type B thoracolumbar burst with incomplete neurologic deficit were enrolled. All of the patients accepted the treatments of posterior indirect reduction and pedicle screw fixation without laminectomy. Clinical and radiologic outcomes were assessed preoperatively and postoperatively.

**Results:**

Operations were performed in a relatively short time without massive hemorrhage. Their neurologic functions were improved by at least one Frankel grade. The average score of American Spinal Injury Association (ASIA) motor increased from 25.4 ± 10.8 to 42.1 ± 10.5, and the recovery rate of the ASIA score was also increased. The pain level was relieved for all the patients. The local kyphosis angle was reduced from 25.9° ± 3.4° to 6.9° ± 2.2° (*P* <0.05) and remained 7.9° ± 2.0° (*P* > 0.05) at the latest follow-up. After the operation, the mean vertebral canal diameter increased from 5.5 ± 1.3 to 11.1 ± 2.2 mm (*P* < 0.05) and the mean canal stenosis index increased from 32.9 ± 7.8 to 84.8 ± 7.3 % (*P* < 0.05). There were no serious complications and fixation failures during follow-up.

**Conclusion:**

Denis type B thoracolumbar burst fractures with incomplete neurologic deficit can be effectively treated by posterior indirect reduction and pedicle screw fixation without laminectomy.

## Introduction

Burst fractures are defined as fracture or comminution of both the anterior and middle columns with retropulsion of bony fragments into the spinal canal, which may occur regardless of the posterior column failure. Most of these fractures occur in the thoracolumbar region and are associated with kyphotic deformity. Subsequent neurologic injury has been reported to occur in 30 to 90 % of patients with thoracolumbar burst fractures, owing to the disruption of the conus medullaris or proximal regions of the spinal cord [1–3].

The purpose of the treatment is to stabilize the restoration, align the spine, and decompress neural elements. Operative management has been widely accepted as the form of management of the majority of thoracolumbar burst fractures, especially when coupled with a neurologic deficit. Various surgical procedures have been applied including anterior and posterior decompression and fusion [4–7]. Anterior approaches are used to achieve direct decompression of spinal canal compromise [6, 8–11]. However, it is necessary to resect considerable amount of anterior elements in the surgery, which leads to formidable surgical onslaughts [6, 8, 10, 12, 13]. In contrast, it can offer comparable neurologic outcome by posterior distraction and stabilization through pedicle instrumentation [7, 14–16], although it is less extensive. Some researchers have considered that neurologic recovery benefits from surgical decompression. However, others have had the opposite attitude to the role of surgical decompression for improvement and restoration of neurologic functions [9, 17], supporting operative treatment for the thoracolumbar burst patients. Up to now, few reports have been published on the posterior indirect reduction and fixation without laminectomy for the thoracolumbar burst patients with incomplete neurologic deficit. Here, we report the experience and evaluate the efficacy of the procedure based on 36 Denis type B thoracolumbar burst patients with incomplete neurologic deficit using posterior indirect reduction and fixation without laminectomy. The purpose of this study was to evaluate the efficacy of the procedure for thoracolumbar burst fractures with incomplete neurologic deficit.

## Materials and methods

From March 2008 to May 2012, 9 women and 27 men aging from 20 to 56 years were enrolled. The average age was 35.2 years old. The level of the injury was T11 in 3 patients, T12 in 6, L1 in 19, and L2 in 8.

**Fig. 1:**
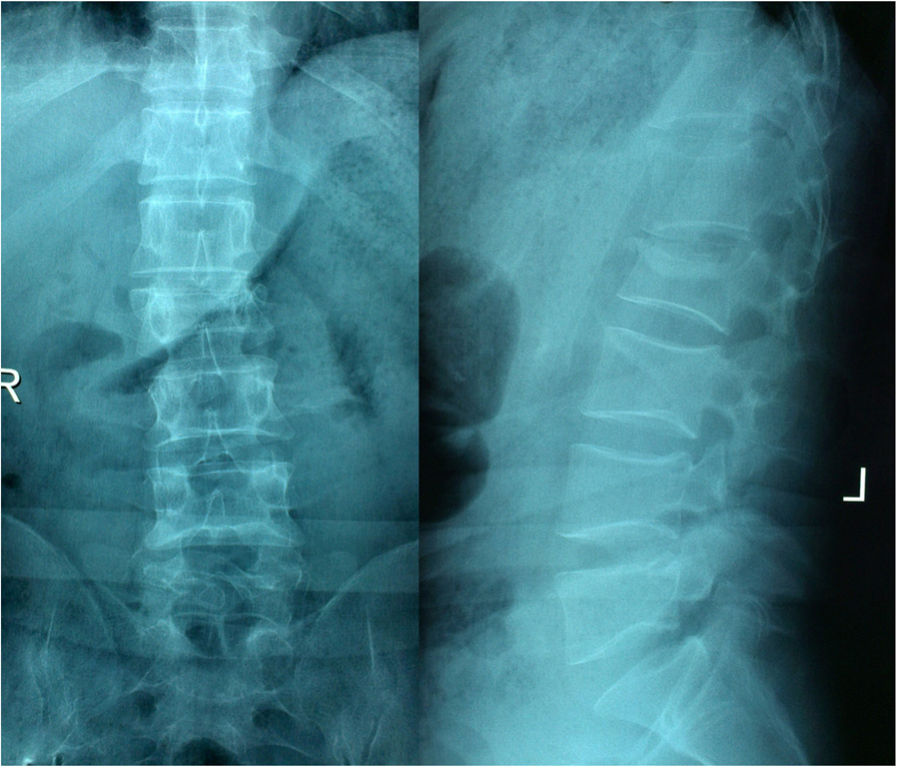
Anteroposterior and lateral radiographs indicate a burst fracture of L2

**Fig. 2:**
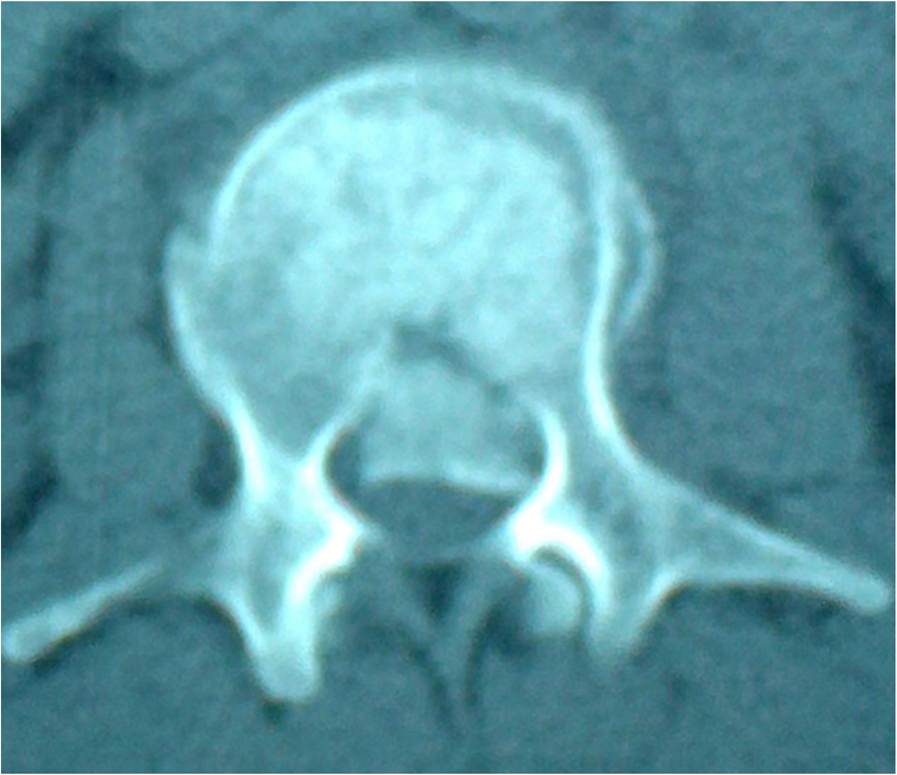
CT scan shows obvious retropulsion of bony fragments into the spinal canal

The inclusion criteria were the single-level thoracolumbar (T11–L2) Denis type B (superior end plate) burst fracture confirmed with plain radiographs and computed tomography, incomplete neurologic deficits (Frankel B–D) independently confirmed with full neurologic examination by at least two experienced spinal surgeons at the time of admission, and admission to our hospital within 3 days after injury (Figs. 1 and 2). Patients were excluded when meeting at least one of the following criteria: 1) complete neurologic deficits (Frankel A), 2) ongoing compression of the neural elements, 3) pathologic or osteoporotic fractures, 4) pre-existing spinal deformity, 5) combination of other site fractures or medical condition requiring intervention, and 6) a history of previous spine surgery.

Anterior–posterior and lateral radiographs and computerized tomography (CT) were obtained for all patients as preoperative and postoperative radiologic evaluation. Postoperative films were taken on the day after the operation and subsequently during outpatient follow-up. Follow-up was usually scheduled at 4 weeks after hospital discharge and at 3- to 6-month intervals thereafter in the first year. The anteroposterior canal dimension at the maximum area of the retropulsed osseous fragment was compared with the average diameter of one level above and one level below, and was expressed as canal stenosis index. The kyphosis angle was measured on lateral radiographs using the Cobb method preoperatively, postoperatively, and during later follow-up.

The neurologic deficits of the patients were assessed according to Frankel’s grading system and the scoring system of the American Spinal Injury Association (ASIA) [18, 19]. The recovery rate of the ASIA score was defined as [ASIA score at the time of the latest follow-up − initial ASIA score]/[50 − initial ASIA score]. The back pain intensity was described by measuring the visual analogue scale (VAS). Compared with the preoperative status of the patients, postoperative neurologic results and back pain were evaluated by two experienced spine surgeons. Any perioperative and follow-up complication was recorded.

The study was approved by the Institutional Ethics Committee of Soochow University. All the patients agreed to participate for the research and signed the written notice of informed consent.

Posterior indirect reduction and pedicle screw fixation (DePuy Synthes Spine Corp., Raynham, MA) were conducted for all the patients. They accepted single surgeries after an average of 3.5 days (ranging from 1 to 7 days) of the initial trauma.

**Fig. 3:**
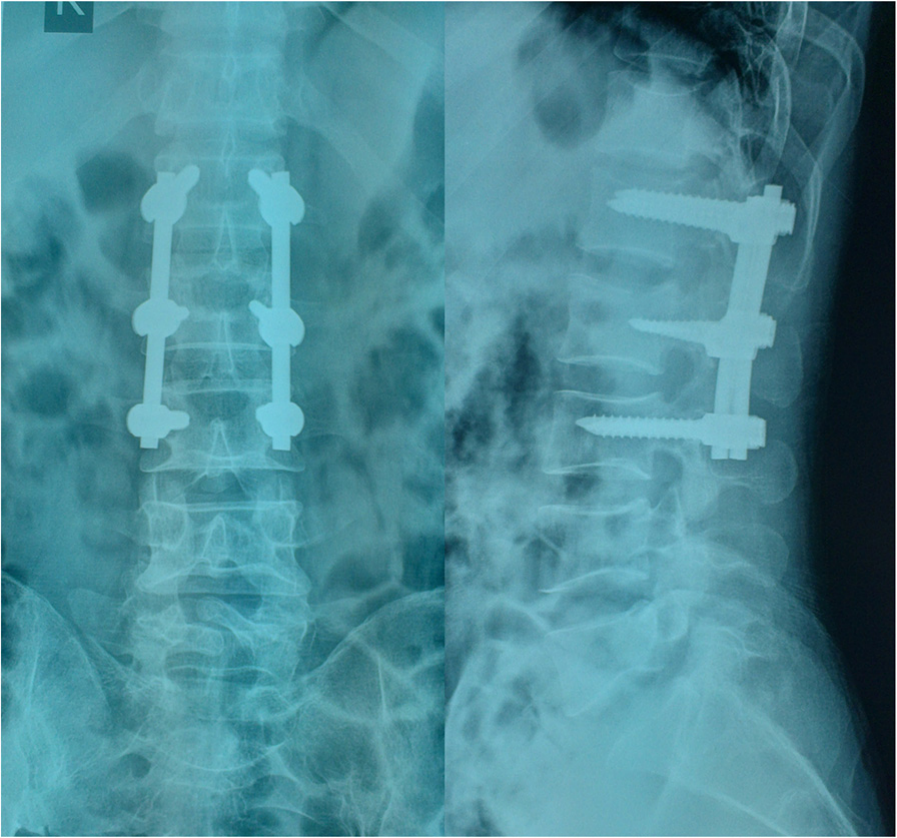
Kyphotic deformity was corrected after posterior indirect reduction and fixation without laminectomy

**Fig. 4:**
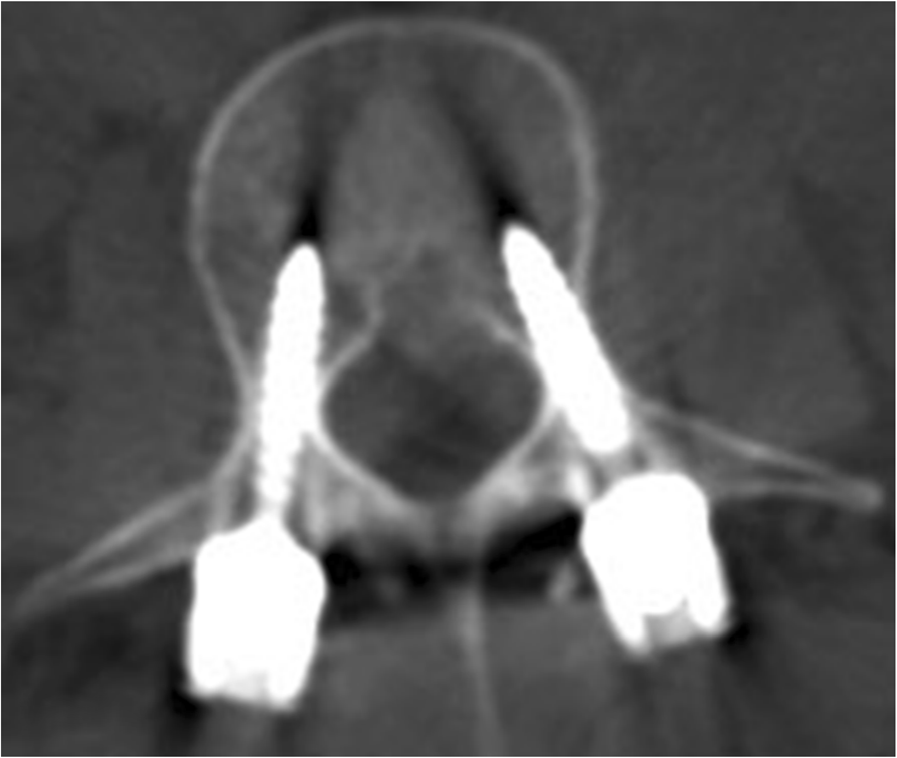
CT scan demonstrates reduction of canal encroachment after operation

Indirect reduction by “ligamentotaxis” was performed in all the instances. The reduction procedure comprised the following steps: 1) correction of the kyphosis, 2) lordotic distraction for further reduction of the vertebral height and intracanal fragment, and 3) rigid locking of all nuts. In general anesthesia, the back relaxed completely. The abdomen of the patient was suspended by pillows under the chest and bilateral ilia. And then the palm of the surgeon pressed the spine gently. Such postural reduction could recover the vertebral height effectively. The right placement of the pedicle screws and the adequate reduction of the fracture can be ensured by C-arm fluoroscopy during operations. When a continuous and smooth posterior vertebral body line of the injured vertebrae appeared in a standard lateral view of the thoracolumbar spine, similar to below and above the vertebrae, the spinal canal bone fragment was considered to be satisfactorily reduced. No laminectomy or bone graft fusions were performed for these patients (Figs. 3 and 4).

After the operation, the patients had a rest in the first 3 days. They were encouraged to stand and walk, when they could turn over in bed without any pain. When they had a regular neurological status, they were allowed to exercise gradually. Normal activity was prohibited in the 6 months after surgery. Generally, the pedicle screw system was taken out after 1 year of fixation.

Data was presented as means ± standard deviation. Nonparametric Frankel scale was compared using the Mann–Whitney *U* test. Other parametric variables were compared by one-way ANOVA. A *P* value <0.05 was considered statistically significant.

## Results

The mean surgical time was 119.2 ± 21.3 min (ranging from 90 to 160 min). The amount of blood loss was 322.2 ± 67.0 ml, ranging from 250 to 500 ml. No major complications were noted perioperatively in any patients. The average duration of stay in the hospital was 12.4 ± 1.8 days (ranging from 9 to 15 days). The averaged follow-up was 32.8 ± 11.6 months. Instrumentation failure, misplaced pedicle screws, or pseudarthrosis had not been observed during the follow-up.

According to Frankel’s grading scale, 9 (25.0 %) patients had Frankel B, 12 (33.3 %) patients had Frankel C, and 15 (41.7 %) patients had Frankel D before operation.

Neurologic deterioration did not occur in any patient after the surgery. During the latest follow-up, patients exhibited neurologic improvement. The median improvement was 1.1 grades in Frankel’s scale. It was found that their neurologic status is significantly improved in pre-operation and the end of the follow-up by nonparametric comparisons. Among the 9 patients who presented Frankel B preoperatively, 7 of them improved to Frankel C and 2 of them improved to Frankel D after operation. Ten out of 12 patients of Frankel C improved to Frankel D, and 2 of them recovered completely (Frankel grade E). Patients with Frankel D had the best recovery. All Frankel D patients attained normal neurologic status. The mean ASIA motor score had improved in all 36 patients (from 25.4 ± 10.8 to 42.1 ± 10.5) with an increase in the recovery rate of the ASIA score (Table 1).

**Table 1:** Neurologic recovery (*n* = 36)

	Preoperative	Postoperative
Frankel scale grade^a^		
A	0	0
B	9	0
C	12	7
D	15	12
E	0	17
ASIA score*	25.4 ± 10.8	42.1 ± 10.5

*ASIA* American Spinal Injury Association

*Statistically significant: *P* < 0.05

^a^The median improvement was 1.1 grades in Frankel’s scale

Most patients suffered from severe pain after the injury. The mean preoperative VAS score was 7.0 ± 0.9. Substantial pain relief was attained in all the patients. Postoperatively, the mean VAS score was reduced to 1.8 ± 0.7 seven days after the procedure. The effect of pain control was persistent. The majority of patients achieved pain-free status during the follow-up.

### Radiologic evaluation

The average local kyphosis angle of the initial stage was 25.9° ± 3.4°. After surgery, significant kyphosis correction was found, where the local kyphosis angle was reduced to 6.9° ± 2.2° immediately and remained 7.9° ± 2.0° at the latest follow-up. Although some patients had mild loss of reduction during the follow-up period, the difference did not change significantly at any time interval. The mean vertebral canal diameter increased from 5.5 ± 1.3 mm pre-operation to 11.1 ± 2.2 mm post-operation. The mean canal stenosis index was 32.9 ± 7.8 % and 84.8 ± 7.3 % before and after the operation, respectively. Decompression was well-maintained over time. The mean canal stenosis index was 94.0 ± 3.5 % at the latest follow-up (Table 2).

**Table 2:** Correction of kyphotic deformity and vertebral canal (*n* = 36)

	Preoperative	Postoperative	*P*
Kyphosis angle (°)	25.9 ± 3.4	6.9 ± 2.2	<0.01
Vertebral canal diameter (mm)	5.5 ± 1.3	11.1 ± 2.2	<0.01
Canal stenosis index (%)	32.9 ± 7.8	84.8 ± 7.3	<0.01

### Discussion and analysis

Most of thoracolumbar burst fractures are associated with the retropulsion of a fragment from the posterior cortex of the vertebral body into the spinal canal and kyphotic deformity. Traumatic injuries are a frequent cause of compressive neurologic syndromes. Subsequent neurologic injury has been reported to occur in 30 to 90 % of patients with thoracolumbar burst fractures [1–3]. These patients present with incomplete neurologic dysfunction or complete neurologic dysfunction. Complete neurologic dysfunction implies severe traumatic injury of neural tissues and poor recovery. In this study, only patients with incomplete neurologic deficit were enrolled considering potential minimal change of complete neurologic dysfunction.

It is controversial for the treatment of thoracolumbar burst fractures. The treatment depends on the individual characteristics of the fracture, with the options including conservative treatment such as bed rest alone, closed reduction and functional bracing, and open reduction and internal fixation. There is no consensus with regard to which method should be selected for the treatment of fractures of varying severity. Although systematic reviews of the effectiveness of nonoperative or operative treatment have failed to demonstrate the superiority of one approach over the other [5, 20–22], operative means have been assumed to facilitate early painless mobilization by providing substantial stabilization of the fractured segment of the spine. Various surgical procedures have been applied including anterior and posterior decompression and fixation. Anterior instrumentation provides the direct decompression and sufficient kyphosis reduction. However, the anterior approaches usually lead to significant surgical onslaughts that may lead to visceral and vascular injury with greater chances [6, 8, 10, 12, 13]. Percutaneous pedicle screw fixation is a minimally invasive operation intended to bolster and support the spinal column. Although it is a good option for thoracolumbar burst fractures, the cost of instrumentation is still high.

In contrast, posterior distraction and stabilization using pedicle instrumentation is less extensive and offers comparable neurologic outcome [7, 14–16]. Posterior indirect reduction and fixation with laminectomy is being selected more often when operative intervention is chosen for the thoracolumbar burst fracture with neurological deficiency. However, some reports supported the opinion that decompression of neural tissue has not produced a significant neurologic improvement [9, 17]. They questioned whether laminectomy is an essential therapeutic strategy for functional recovery. In their opinions, static canal compromise cannot reflect the dynamic trauma progress. Damage to the cord occurs at the time when the bone fragment is retropulsed with great energy into the spinal canal, and the canal compromise shown on static images after the accident will not be able to represent this dynamic fracture process. The spinal canal is almost certainly less narrowed than when the cord is impacted [23–26].

There are still concerns about long-term change of the narrowed spinal canal and risk of continuous compression of the spinal cord. Leferink et al. [16] studied changes of the spinal canal during operative treatment and follow-up. It is found that bony encroachment of the vertebral canal was seen preoperatively in 76.5 % of the patients, postoperatively in 18.4 %, at 9 months in 8.2 %, and at 2 years in 2.4 %. Numerous studies have discovered the spontaneous remodeling of the spinal canal during the course of treatment, with or without decompression, and degree of canal stenosis reduced with time [16, 27–31]. Johnsson et al. [12, 32] declared that even if surgery was not undertaken, the bone fragments would remodel, resulting in the gradual clearance of narrowed canal as adequate as that achieved by some surgical decompression. Therefore, incomplete reduction of retropulsed bone fragments from the injured vertebral body was not associated with delayed neurologic deficit or deterioration. It was concluded that removal of intraspinal fragments was no longer necessary.

Based on the above theory, we used instrumental decompression of the spinal canal at the thoracolumbar junction in an indirect way, a mechanism known as ligamentotaxis. If the longitudinal ligaments are not (completely) disrupted, distraction and anti-kyphosis can achieve a reduction of bony fragments, widening the spinal canal of the injured spine. The effect of the forces conducted via the attachment of the annulus to the end plates by instrumental and postural anti-kyphosis reduction will add to the restoration of the spinal canal wall. When a continuous and smooth posterior vertebral body line of the injured vertebrae appeared in a standard lateral view of the thoracolumbar spine, similar to below and above the vertebrae, the spinal canal bone fragment was considered to be satisfactorily reduced. Significant kyphosis correction and retropulsed bone reduction were achieved in the study, avoiding the open manipulation of the bony fragment. All patients exhibited neurologic improvement by at least one Frankel grade, contributing to the average neurologic recovery of 1.1 Frankel grades. Our preliminary data indicates that operation time for posterior fixation and decompression in an indirect way ranges from 90 to 160 min (mean 119.17 ± 21.3 min), which is statistically shorter than that for laminectomy. Also, blood loss (ranging from 250 to 500 ml) in all cases was recorded at a lower level compared with open surgical decompression.

## Conclusions

Our study demonstrated that thoracolumbar burst fractures with incomplete neurologic deficit could be treated by posterior indirect reduction and fixation without laminectomy. The selected patients with incomplete neurologic deficit did benefit from the surgery. This strategy might be a good alternative for Denis B fractures. However, it is not our intention to argue whether surgical decompression of burst fractures should be done, since treatment strategies need to be individually designed depending on the special details of patients. We recommended that early surgical decompression should be taken to improve the function of the patients with neurological deficits secondary to thoracolumbar burst fractures under the condition of the ongoing compression of the neural elements. However, we will further our study for more techniques such as vertebroplasty/kyphoplasty, combined anterior and posterior fixation, and percutaneous pedicle screw fixation about this issue. Hopefully, a series of our researches will offer more useful information to spine surgeons.
